# Experimental Study of Slag Changes during the Very Early Stages of Its Alkaline Activation

**DOI:** 10.3390/ma15010231

**Published:** 2021-12-29

**Authors:** Vlastimil Bílek, Petr Hrubý, Valeriia Iliushchenko, Jan Koplík, Jakub Kříkala, Michal Marko, Jan Hajzler, Lukáš Kalina

**Affiliations:** Faculty of Chemistry, Brno University of Technology, Purkyňova 118, 61200 Brno, Czech Republic; xchrubyp@fch.vut.cz (P.H.); xciliushchenko@vutbr.cz (V.I.); koplik@fch.vut.cz (J.K.); xckrikala@fch.vut.cz (J.K.); xcmarkom@fch.vut.cz (M.M.); xchajzlerj@fch.vut.cz (J.H.); kalina@fch.vut.cz (L.K.)

**Keywords:** alkali-activated slag, activator, hydration kinetics, pore solution, scanning electron microscopy, BET surface, morphology, hydration products, setting

## Abstract

The very early stages of alkaline activation of slag control its rheology and setting, but also affect its hydration, which occurs later. Simultaneously, these parameters are dictated by the nature and dose of the alkaline activator. Therefore, we investigated and compared the changes in slag particles (SEM, BET, laser diffraction), as well as in the pore solution composition (ICP–OES), pH, and conductivity, of alkali-activated slag (AAS) pastes containing the three most common sodium activators (waterglass, hydroxide, and carbonate) and water during the first 24 h of its activation. To ensure the best possible comparability of the pastes, a fairly nontraditional mixture design was adopted, based on the same concentration of Na^+^ (4 mol/dm^3^) and the same volume fraction of slag in the paste (0.50). The results were correlated with the pastes’ hydration kinetics (isothermal calorimetry), structural build-up (oscillatory rheology), and setting times (Vicat). Great differences were observed in most of these properties, in the formation of hydration products, and in the composition of the pore solution for each activator. The results emphasize the role of the anionic groups in the activators and of the pH, which help predict the sample’s behavior based on its calorimetric curve, and offer data for further comparisons and for the modelling of AAS hydration for specific activators.

## 1. Introduction

The search for ways to reduce the CO_2_ footprint in the cement and building industry is a very current topic. One possibility is the use of alternative binders, including alkali-activated materials (AAMs), which can reduce CO_2_ emissions by up to 80% compared to ordinary Portland cement or clinker-based materials [1]; however, this strongly depends on the mix design [2]. Although AAMs have been found to be advantageous in terms of their resistance to chemical attacks [3,4,5,6,7,8], frost resistance [1,9,10], resistance to high temperatures and fire [11,12,13,14], and their highly dense interfacial transition zone [15], their wider practical use remains a challenge. For low-calcium systems such as alkali-activated fly ash (AAFA), elevated temperatures are needed to ensure their hydration, while for alkali-activated slag (AAS)—as the main representative of high-calcium systems—excessive drying and autogenous shrinkage [16], and sometimes rapid setting [17,18], are a problem. Therefore, both AAS and AAFA are often blended to overcome their individual disadvantages [19,20,21,22,23]. However, the common issue for both systems is the use of plasticizing additives to control their rheology, which is associated with their chemical stability and miscibility in the activating solution, as well as their competitive adsorption with activator anions [24].

The nature and dose of the alkali activators dictate not only the rheology of AAMs but also their hydration kinetics, and thus, their setting and hardening processes and strength evolution, as well as their durability and other properties; thus, increasing the complexity of AAM technology. While the long-term properties of AAMs with various activators are quite well documented and understood, the processes that occur during the first minutes and hours of hydration have not yet been explored sufficiently. The key parameter is the dissolution of the amorphous aluminosilicate phase, leading to oversaturation of the pore solution and the precipitation of hydration products. For granulated blast furnace slag, the dissolution rate is relatively high even at room temperature, while the glassy phase of fly ash dissolves slowly, from which the requirement of elevated temperatures (usually 60–95 °C is used [23,25,26]) originates. It is known [27,28] that the dissolution of slag glass is strongly dependent on the disorder of the aluminosilicate framework due to its alkali content (K^+^, Na^+^)—especially alkaline earth metal cations (Ca^2+^, Mg^2+^). This can be expressed nicely in terms of the ratio of non-bridging oxygens to those that are tetragonally coordinated, which typically reaches values of 1.7 to 2.0 for industrial slags [29,30]. Additionally, the reactivity of the slag is significantly influenced by the presence of minor elements, of which, network modifiers such as Ba and Sr increase the reactivity of the slag, while others such as Ti, Ce, Cr, V, and Zr have a dramatic adverse effect [31]. The dissolution rate also depends on the shape of the particles and increases with the increasing irregularity of the particles [30].

In addition to the above-mentioned aspects, the activator composition and concentration are another key parameter affecting the dissolution of the slag, as well as the composition of the hydration products, the rate of their formation, etc. One tool for the reasonable prediction of which solid products will be able precipitate (if any), i.e., to assess the under/supersaturation of the solution with respect to the given solid phase, is the so-called effective saturation index, described elsewhere [30,32,33]. The formation of reaction products can also be modeled using GEM-Selektor, which is a software that works based on the minimization of Gibbs energy [34,35].

Out of the most common alkali activators, the simplest hydration process can be expected for (sodium) hydroxide, in which both Na^+^ and OH^−^ act as catalysts—especially in the dissolution stages of hydration [18]. Na^+^ also typically constitutes various aluminate–silicate hydrates to form N-A-S-H, C-A-S-H or (C,N)-A-S-H [36], which are the main hydration products in AAMs. While the N-A-S-H gel has a rather three-dimensional structure without chemically bounded water [18], the structure of C-A-S-H is rather layered [37]. This allows the interaction of numerous non-bridging oxygens from the silicate chains and calcium counterions with the surrounding species—for example, with various polymers that modify the properties of C-A-S-H and can even form advanced inorganic/organic nanocomposites [38,39,40].

The anionic groups of sodium carbonate and sodium silicate activators are present in high concentrations from the beginning of the mixing process. This affects the dissolution of the aluminosilicate precursor; for example, the leaching of Si from slag is hampered by a high concentration of silicates [33], but also results in the formation of various early hydration products prior to the main hydration stage. This in turn significantly affects the properties of AAS in the very early stages—typically its rheology and setting time, as well as the further hydration processes. Some of these products are not stable, and with ongoing hydration they dissolve and form less soluble solid phases, such as gaylussite in sodium carbonate-activated slag [41]. However, these initial stages of hydration have not yet been adequately studied, especially in terms of the comparison of various activator compositions.

Therefore, this paper deals with changes in slag particles starting from the end of the mixing process up to 24 h of the hydration stage by means of their size, morphology, and the composition of the pore solution, in the context of their hydration kinetics—as determined by isothermal calorimetry—and their early mechanical properties (rheology, setting). These aspects are compared for the most common alkali activators, namely, sodium waterglass, sodium hydroxide, and sodium carbonate.

## 2. Materials and Methods

### 2.1. Materials and Mixing Procedure

Ground granulated blast furnace slag with a density of 2.88 g/cm^3^ and a Blaine fineness of 400 m^2^/kg of Czech production (supplied by LB Cemix, s.r.o., Štramberk, Czech Republic) was used as a starting aluminosilicate precursor. Its chemical composition as determined by X-ray fluorescence is given in Table 1. From the mineralogical viewpoint, it consisted mainly of an amorphous phase (~70%), about 20% akermanite, and minor amounts of calcite, quartz, and merwinite.

Three different activators were used to prepare the activating solutions: sodium waterglass (Vodní sklo, a.s., Brno, Czech Republic) with a silicate modulus (SiO_2_ to Na_2_O molar ratio) of 1.89 and 47.92% dry matter; 49.61% sodium hydroxide solution (Carl Roth GmbH + Co. KG, Karlsruhe, Germany); and powdered sodium carbonate, with analytic-grade purity (Lach-Ner, s.r.o., Neratovice, Czech Republic). From these activators, activating solutions with the same concentration of 4 mol Na^+^/dm^3^ were prepared in advance to sample preparation. At the same time, the silicate modulus of the waterglass was reduced to 1.5 by a solution of sodium hydroxide. The activator concentration was one of the two parameters that described the composition of the prepared pastes; the second was the volume fraction of slag in the paste set to 0.50 for all cases, except for additional experiments carried out with a volume fraction of slag of 0.10. The activator type was also used as a part of the sample label, namely, SH for sodium hydroxide, SWG for sodium waterglass (silicate modulus of 1.5), and SC for sodium carbonate. The corresponding AAS pastes were then marked as SHAS, SWGAS, and SCAS. For comparison, slag suspension in water (WAS) was also prepared at the same volume fractions (0.50 and 0.10).

Paste samples were prepared by adding slag to the activating solution and the mixture was homogenized for three minutes using a hand mixer. Then, it was placed in polypropylene vials and sealed with a screw cap until the various characterizing procedures and analyses were carried out, as described in the following sections—i.e., 5 min, 30 min, 60 min, 120 min, 5 h, and 24 h since the mixing.

### 2.2. Paste Characterization

The effect of the activator on the hydration kinetics was assessed using a TAM Air isothermal calorimeter (TA Instruments, New Castle, DE, USA) at 25 °C. Based on the obtained results, the above-mentioned timing of the paste characterization was planned. The setting process was determined using the Vicat needle test based on EN 196-3, but the sample was poured with paraffin oil to prevent drying and dilution of the activator if water was used.

The structural build-up of the pastes prior to setting was monitored using a rotational rheometer DHR-2 (TA Instruments, New Castle, DE, USA) equipped with a stainless-steel vane-in-cup geometry in an oscillation mode with a frequency of 10 rad/s. The strain amplitude within the linear viscoelastic region (LVR), determined in advance using the strain sweep test, was set to 0.001%. Before each test, a gentle pre-shear was carried out at 10 s^−1^ for 10 s. The measurement started 5 min after the first contact of the slag with the activating solution. To prevent drying, the measuring cell was covered with a solvent trap.

### 2.3. Isolation of the Pore Solution and Its Characterization

The isolation of the pore solution depended on the physical state of the paste, as follows. Sufficiently liquid pastes, prior to their setting, were centrifuged at 3600 rpm for 5 min. Consequently, the supernatant was filtered through a nylon syringe filter (0.45 μm) and 1 mL of it was diluted in a plastic volumetric flask with demineralized water. The pore solutions of the set or hardened specimens were obtained using the press-die method and then processed in the same manner as those of the centrifuged samples. The conductivity and pH values of the diluted pore solutions were determined using a SevenCompact Duo S213 equipped with InLab 731 and InLab Expert Pro (Mettler Toledo GmbH, Greifensee, Switzerland). Elemental compositions (concentration of Na, Si, Al, Ca, Mg, K, Fe, Ti, Mg) were analyzed using optical emission spectroscopy with inductively coupled plasma (ICP–OES).

### 2.4. Isolation of the Solids from the Pastes

As with the isolation of the solids, the isolation of the pore solution depended on the physical state of the paste. During the very early stages, the solid content (predominantly slag particles) was obtained by diluting the paste by an excess of demineralized water at the given time, then its vacuum filtration, washing it with water, and, finally, with acetone to remove any resting water and to facilitate drying. Drying was carried out at 40 °C. In the case of waterglass, the filtration was extremely slow, which is related to its suspension stability (high magnitude of zeta potential [42]), so it had to be centrifuged after dilution with water—the supernatant containing the finest fractions that had not been discarded—and the sample was diluted again with water before the following filtration.

At later stages, typically after setting, where the diluting of the pastes was impossible, the samples were broken and pieces of the paste were immersed in water and then in acetone to stop their hydration, and then dried again at 40 °C. Washing with water prior to immersion in acetone was carried out to prevent the precipitation of solid products induced by organic solvents that occurs in some cases—typically for waterglass [43].

#### Analyses of the Solid Phase

The particle size distribution (PSD) of the samples without macroscopically observable aggregates was determined using the laser diffraction method using Helos/KR instrument (Sympatec GmbH, Clausthal-Zellerfeld, Germany) equipped with objective R3 (for particle sizes of 0.5–175 μm).

At the selected hydration times, the BET surface area was determined using the nitrogen adsorption analyzer NOVA 2200e (Quantachrome Instruments, Boynton Beach, Florida, USA). Before measurement, the samples were degassed at 40 °C for 20 h, as inspired by the recommended approach of Mantellato et al. [44] for anhydrous cement samples.

The morphology of the slag particles and the presence of hydration products were assessed using scanning electron microscopy using the Carl Zeiss EVO LS 10 instrument (Carl Zeiss Nts, LCC, Peabody, MA, USA) in the mode of secondary electrons at various magnitudes, after the samples were coated with gold. The acceleration voltage was set to 15 kV.

## 3. Results and Discussion

### 3.1. Hydration Kinetics

**Figure 1:**
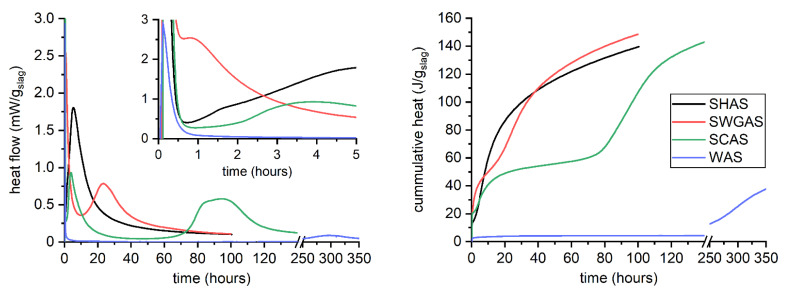
Hydration kinetics of slag activated with sodium hydroxide (SHAS), sodium waterglass with SiO_2_ to Na_2_O molar ratio equal to 1.5 (SWGAS), sodium carbonate (SCAS), and water (WAS), recorded by means of isothermal calorimetry.

The effect of the activators at the same concentration of 4 mol Na^+^/dm^3^ is shown in Figure 1. The general calorimetric response for each alkali activator was in agreement with those reported by Shi and Day more than 25 years ago [45], and more recently by other authors [33,46,47,48,49,50,51,52,53]. All pastes showed an initial peak just after placing the sample in the calorimeter, which originates from the wetting and initial dissolution of the slag grains and the energy absorbed by the sample during the mixing. Unlike AAS pastes mixed by hand mixer for three minutes, water was mixed only by hand with a spatula inside the vial, so its remarkably different initial peak is not affected solely by the different composition of the liquid, but also significantly by the mixing regime.

Then, the calorimetric response was strongly affected by the activators’ nature. SHAS did not show an induction period [33,53] and the second peak started just after the initial peak, indicating large amounts of precipitated hydration products—especially the C-A-S-H gel. In the case of SCAS and SWGAS, a second peak adjacent to the initial peak also occurred. This originated mainly from the reaction of anions from the activator with dissolved species from the slag—particularly calcium ions. For SWGAS, the resulting product is sometimes called primary C-S-H [43,45] or is referred to as the gelation of the activator [54,55], while for SCAS it is a manifestation of the precipitation of carbonates, e.g., calcite or gaylussite [41,45,53,56].

The last peak, responsible for most of the released hydration heat, was again related to the massive precipitation of hydration products. If the reaction product corresponding to the second peak is called primary C-S-H, this third one can be called secondary C-S-H [49]. Before this third main hydration peak occurred, a distinguishable induction period occurred. For SWGAS, the induction period was attributed to the slowing of slag dissolution, due to the high concentration of dissolved Si [33]. This very long induction period is a common issue of SCAS [41,53,56]. At this stage, the dissolution of slag continues, gaylussite converts to calcium carbonate and releases Na^+^, hydrotalcite and zeolitic phases are formed, and the concentration of OH^−^ and Si in the pore solution increases [41]. The main hydration peak was also distinguishable for slag in water after more than 200 h, showing that slag can slowly react with water to reach the hardened state.

### 3.2. Evolution of Binder at Early Stages

Different hydration kinetics and the nature of early hydration products resulted in markedly different evolutions of the initial mechanical properties of the fresh pastes. Table 2 summarizes the results of the initial and final setting time, as determined by the Vicat needle test. The shortest setting times were recorded for SWGAS at 42–67 min, followed by SHAS at 2–4 h—while SCAS setting was markedly longer, with an initial setting time of about 5 h and a final setting time of approximately 20 h. The fast setting of SWGAS corresponds to the second calorimetric peak and is related to the gelation of silicates in SWG. In some cases, possibly for waterglass with a lower silicate modulus, only stiffening of the paste that can be dispersed again by mixing has been reported [43]. In our case, however, it was not possible to fully disperse the paste—even in excess of water—and hence, agglomerates were obtained.

SHAS effectively dissolves slag, including silicates and aluminates, and reaches an oversaturated state—resulting in the precipitation of hydration products and leading to a relatively fast setting of a few hours. In contrast, SCAS paste slowly loses its plasticity rather than setting during the first hours and days (pre-induction and induction period), due to the presence of precipitating carbonates and, possibly, other crystalline products [41,53]. Although the SCAS paste was already set, as determined by the Vicat needle test, it should be noted that the paste could be dispersed well again in excess of water by mixing it with a spatula. Therefore, the penetration of the needle was affected by the physical effects (i.e., an increase in the solid content with more contact points, the consumption of free water to form hydrated solids) rather than by the evolution of the main binding phase (C-A-S-H) between the slag grains. These physical effects were probably supported by the relatively high volume fraction of the slag in the paste (0.50), so that the paste was relatively stiff even in the fresh state.

**Figure 2:**
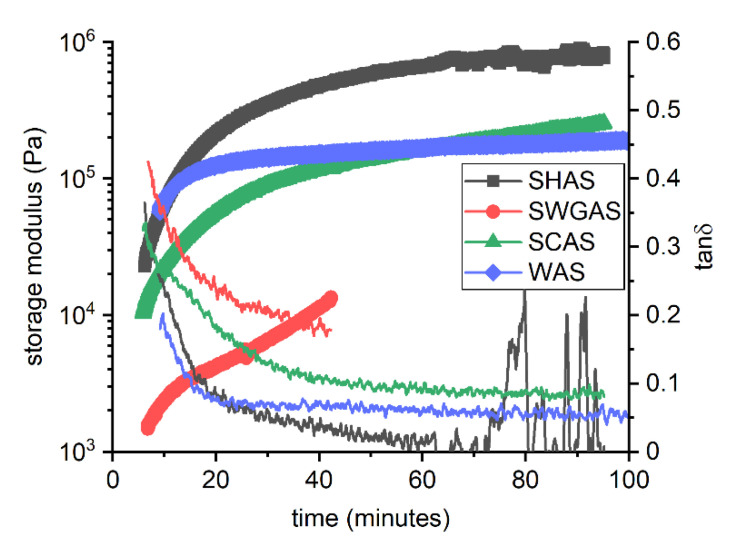
Structural build-up of AAS pastes with different types of activator (SWG—sodium waterglass, SH—sodium hydroxide, SC—sodium carbonate, W—water); line + symbol corresponds to the storage modulus (left y axis); line corresponds to the loss factor (right y axis).

The evolution of the physical properties of these pastes during the first 95 min was determined using a rotational rheometer working in the mode of small amplitude oscillatory shear, with a strain amplitude of 0.001%. The results obtained, expressed in terms of the storage modulus, and the loss factor—defined as the ratio between the loss modulus and storage modulus (tanδ = G″/G′)—are given in Figure 2. For all pastes, the loss factor was significantly lower than one, i.e., the storage modulus prevailed over the loss modulus, so the pastes behaved rather as a viscoelastic solid. Furthermore, all pastes showed noticeable structural build-up—which is in its early stages caused by flocculation of the suspension due to strong attractive forces between particles—and rigidification continued via the formation of the first reaction products [57]. The most rapid precipitation of hydration products was observed for SWGAS, for which the acceleration of the storage modulus increase started at around 25 min of mixing and differed distinctively from the trend during the first minutes, even on the logarithmic scale. The measurement was manually stopped at the time corresponding to the initial setting to ensure that the measuring geometry could still be cleaned relatively easily. The other two pastes did not set within the measurement range (95 min), although for SHAS, disruption of the measured values could be observed after one hour in terms of a slightly scattered storage modulus and a greatly distorted loss factor. This could be related to the high mechanical properties of the sample, as well as to the decrease in its critical strain below the preset value (0.001%). The latter is also an issue for Portland cement pastes [58] and suggests the formation of solid reaction products.

In addition to the structural build-up itself, Figure 2 also shows the impact of the activator on the rheology of AAS pastes. The highest storage modulus, and simultaneously the lowest loss factor, was observed at the beginning of the measurement for SHAS and WAS—indicating the stiffest and the most solid-like structures in the fresh states of these tested pastes. After 15–20 min, the behavior of SHAS differentiates from that of WAS in terms of further stiffening of the SHAS paste, as indicated by the ongoing increase in the storage modulus and the simultaneous decrease in the loss factor. In contrast, by far the lowest storage modulus and the highest loss factor were observed for SWGAS. This is related to the differences in its surface chemistry, induced by the different nature of its activator anions. Usually, slag particles dispersed in water have a slightly negative zeta potential [42,59], which is further shifted to more negative values by the adsorption of negatively charged silicates, and thus, the paste is plasticized, while the zeta potential of SHAS is around zero, resulting in higher rheological parameters such as yield stress [42]. The viscoelastic properties of SCAS are in between those of SHAS and SWGAS, which again can be explained by means of colloidal interactions. The adsorption of carbonate anions onto the slag grains should increase the magnitude of the zeta potential (shift to more negative values) and thus shield attractive interparticle forces. However, silicates can form larger species compared to carbonate anions from Na_2_CO_3_, and can therefore act over longer distances, which may contribute to the observed rheological differences.

**Figure 3:**
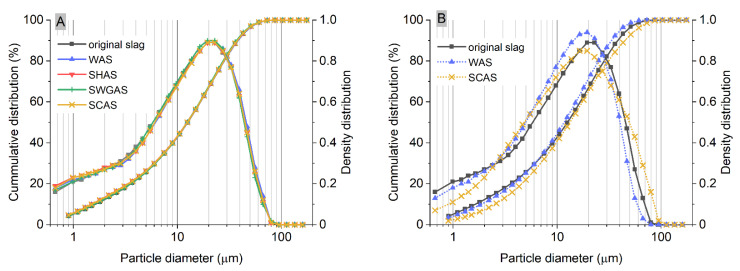
Illustration of the negligible effect of slag dissolution on particle size distribution during the first 30 min, regardless of the type of alkaline activator used for pastes, with a slag volume fraction of 0.50 (**A**), and the effect of a decreased slag volume fraction of 0.10 in SCAS and WAS after 24 h (**B**).

To support the findings mentioned above and to add a more complex perspective concerning the changes in the solid phase and their relation to early mechanical performance, the changes in the slag particles over time were monitored using SEM, BET surface area, and laser diffraction. An example of the results from the latter method is given in Figure 3A, while a numerical summary of the results obtained is given in Appendix A (Table A1). PSD determination was performed only on the visually well-dispersed samples after the addition of water and remixing of the diluted sample by hand with a spatula, i.e., before or during the early stages of the setting. The very short time of hydration was the main reason why significant changes in PSD were not detected, and simultaneously shows that the laser diffraction method used was not a suitable or sufficiently sensitive method for this purpose. Only the SCAS results after 5 and 24 h showed a noticeable decrease in the size of particles contributing to the first 30% or 50% of the volume of all the particles (x30 and x50 in Table A1), which could be related to the precipitation of, e.g., fine carbonate products. On the other hand, the modus, or in other words, the most frequent particle size of all the determined fractions, was roughly constant (22.91 μm in most cases, Table A1) in all samples—including the original slag. This shows that in the first stages of hydration, the coarse particles are not significantly affected by dissolution.

To intensify the dissolution of the slag, pastes with a volume fraction of slag of 0.10 were also prepared and analyzed after 24 h. In this case, the SWG had already gelled across the whole sample within the 24 h, and the SH resulted in a significant increase in hydration products, leading to particle aggregation (which will be shown later); as such, only the SCAS and WAS could be dispersed well in additional water, and thus, were used for PSD determination. The obtained results are given in Figure 3B. For the slag in water, only slight changes were observed, such as a slight decrease in the fine particle content at the lower limit of the objective (around 1 μm) and an increase in the range of 10–20 μm. For the SCAS, the particles around 1 μm almost completely diminished, while high amounts of relatively coarse fractions of more than 50 μm appeared. This is related to the aggregation of particles as a result of the crystallization of carbonate products. 

More sensitive to changes in slag particles is the determination of the BET surface area. This was carried out on samples from the first 30 min of the reaction and then around the setting or at relatively later stages, selected depending on the physical state of the paste. The obtained results are summarized in Table 3. During the first 30 min, the BET surface area slightly increased for all pastes compared to the original slag. One exception was SWGAS after 5 min, whose reduced surface area was related to the discarding of the finest fractions before filtration, as these fractions did not sufficiently sediment—even in the centrifugal field (see Section 2.4). However, this paste showed the most rapid increase in BET surface area, as a significant difference was apparent even after the first 30 min and especially after one hour, i.e., between the initial and final setting time indicated by the Vicat needle (see Table 2), as well as between 60 and 120 min. A noticeable increase in the BET surface area was observed for SHAS after two hours, which is expected, as it again corresponds to the setting period, but simultaneously showed that the level of hydration products was still relatively low. This is in line with the possibility of dispersing the paste in excess of water, as was already seen with the same PSD at the earlier stages of the reaction (Figure 3A). As the precipitation of the hydration products proceeded, a significant increase in the specific surface was observed after 5 h, along with a slight non-reversible aggregation. Although the specific surface of SCAS increased roughly five times after 24 h of reaction compared to the initial stages, the products still had poor binding ability. The very slow increase in the surface area of the BET for the slag in water confirms that it can be expected to slowly react for a long time before setting.

**Figure 4:**
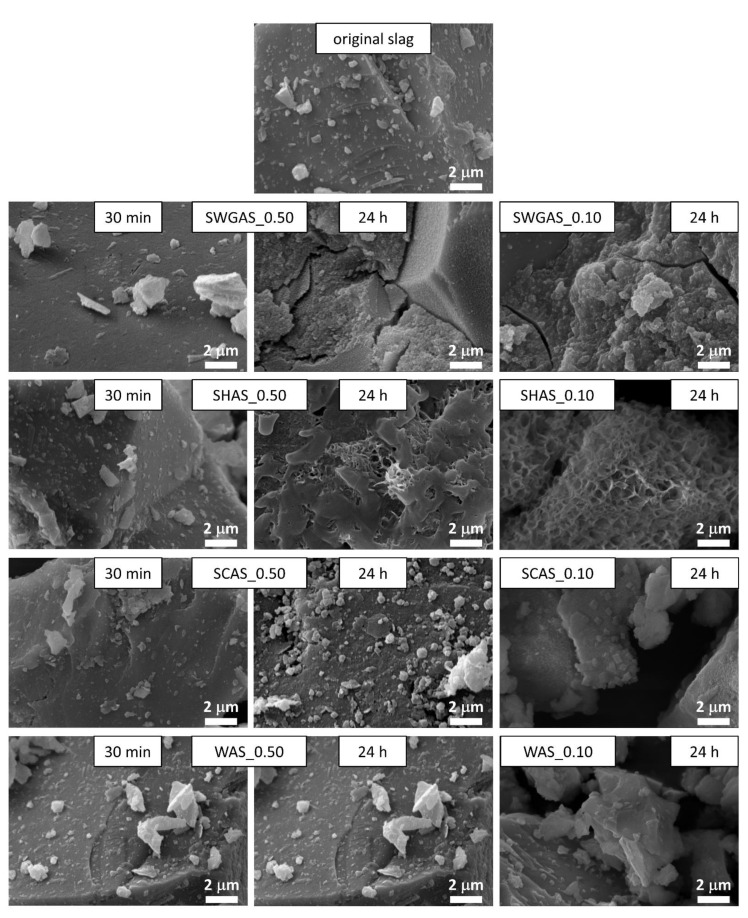
Morphology of AAS paste with different activators (SWG—sodium waterglass, SH—sodium hydroxide, SC—sodium carbonate, W—water) and two different volume fractions of slag in the paste (0.50 and 0.10).

**Figure 5:**
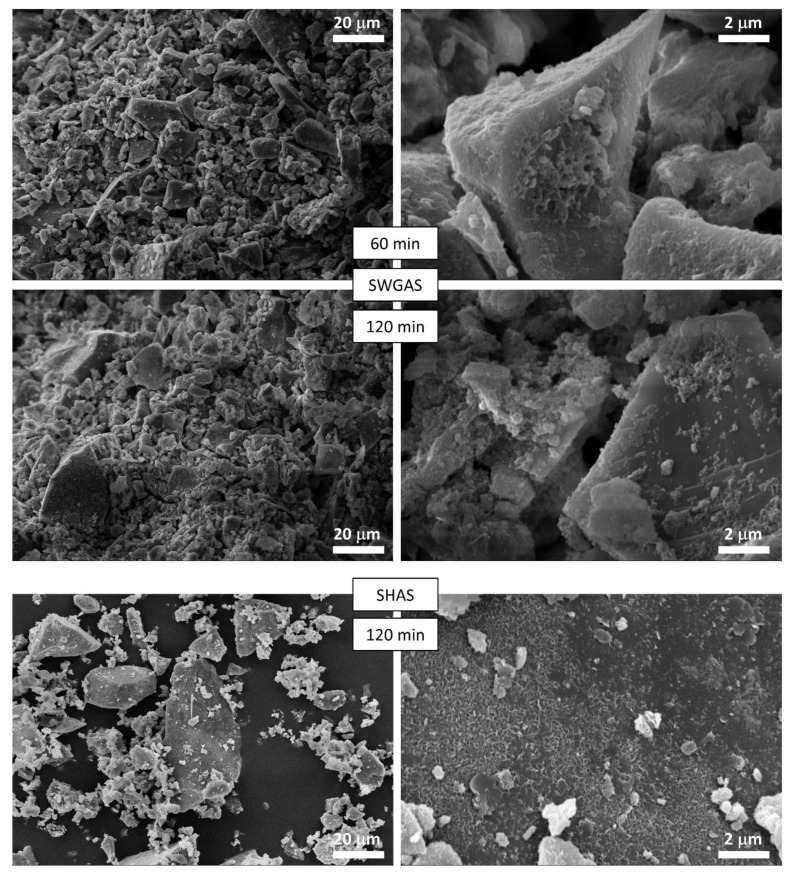
Microstructure of SWGAS and SHAS around their setting periods.

**Figure A1:**
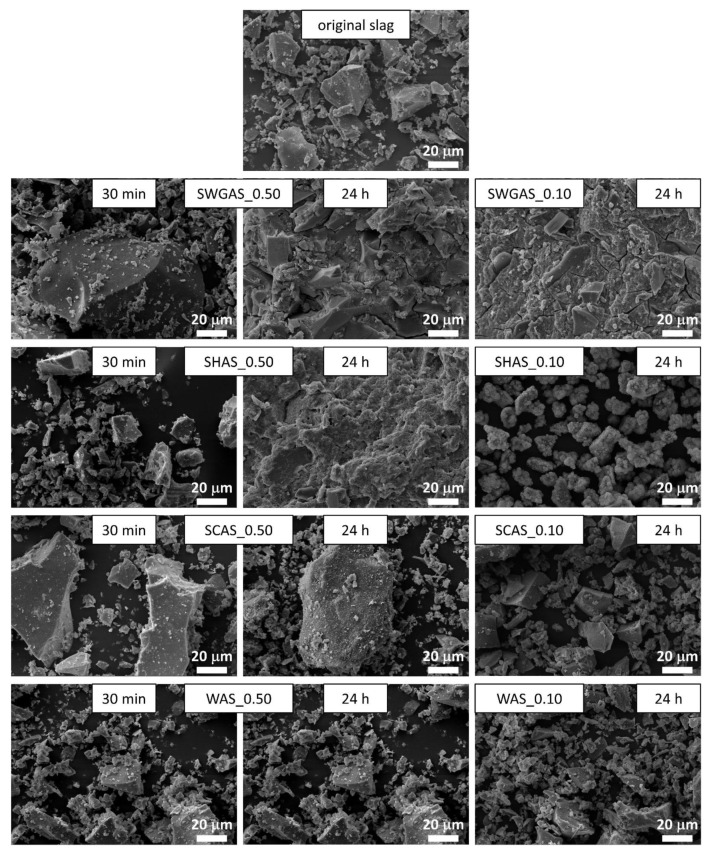
Effect of different alkali activators and water on the microstructure of AAS pastes with a slag volume fraction of 0.50 and 0.10 (supplementary to Figure 4, but 10× lower magnitude).

Until now, the growth of hydration products has been tracked indirectly by methods that characterize the AAS pastes as a whole. To verify these findings, the morphology of the solid particles and the presence of hydration products in the pastes were here observed using SEM. The main summary of the results obtained is given in Figure 4 (higher magnification) and in Appendix B (Figure A1, lower magnification), where the microstructure of the pastes with all activators is given after 30 min and 24 h for a volume fraction of slag of 0.50, and after 24 h for a volume fraction of slag of 0.10. For comparison, the original slag as received from the supplier is included. In addition, Figure 5 presents the microstructure images of SWGAS and SHAS during the setting period.

It can be seen that after 30 min, for the highest magnitude used and regardless of the activator used, there were no obvious changes in the morphology of the slag particles, i.e., no apparent reaction products or visible signs of dissolution on the surface, etc. This corresponds well to the negligible evolution of the surface area of the BET (Table 3) and to previous findings published by Newlands et al. [60], where the thickness of the dissolved glass simulating slag after five minutes was calculated at tens of nanometers. The most rapid setting was determined for SWGAS (see Table 2), which corresponded to SEM images after 1 and 2 h of hydration (Figure 5), where considerable amounts of the first reaction products could be found on the slag particles, binding them together. Their precipitation was also related to a rapid increase in the surface area of the BET (Table 3). The greatest difference occurred at 30–60–120 min, which nicely illustrates how rapid this process is and why rapid setting over tens of minutes is often an issue in SWGAS [61,62]. Based on the results of Palacios et al. [43], both the formation of C-A-S-H and poorly ordered N-A-S-H precipitate in this stage as well as the formation of M-S-H in very low amounts was hypothesized. After 24 h, when the third calorimetric peak reached its maximum (Figure 1), a cracked, but otherwise continuous matrix typical for SWGAS was already present (Figure 5)—indicating a great densification of the microstructure. This decrease in porosity continues on a long-term basis and results in a further increase in mechanical properties [63]. Macroscopically, the same microstructure was also observed for the SWGAS paste with a slag volume fraction of 0.10 (Figure 4), which shows that although the activating solution is in high excess of the slag particles, enough slag is still dissolved during the first 24 h. It is worth mentioning that the continuous matrix of hydration products was formed across the whole paste, despite the fact that the paste was mixed using the overhead shaker for the entire 24 h. This points to a gelation of the silicates occurring in the volume of the pore solution.

When the setting of SHAS occurred, the first reaction products on the surface could be observed to have a noticeably different morphology compared to SWGAS (Figure 5). The great difference between the two pastes also persisted after 24 h (Figure 4), after which, the porous sponge-like structure of the C-A-S-H structure and the footprints of the slag particles could be observed in the SHAS. The observed C-A-S-H corresponds to the ‘outer product’ formed in SHAS during the first days of hydration around the slag particles, while the ‘inner product’ forms at later stages and is located at the site of the original slag grain [63]. The outer product has previously been observed even during late stages on polished samples [63] as well as on fracture surfaces [64], i.e., the same as in the present study. The formation of the inner product does not result in the significant refinement of coarse pores, and therefore, the later-stage strengths of the SHAS are lower compared to those of the SWGAS [63]. In addition to C-A-S-H, hydrotalcite plates are usually found in SEM images [63,64], which were not observed in this work. This can be explained by the relatively low Al content in the slag, while larger hydrotalcite plates are favored by higher Al content [63]. Similar to the SWGAS, the decrease in the volume fraction of the slag to 0.10 in the SHAS did not significantly change the morphology of the hydration products, but individual agglomerates of particles with a surface completely coated with hydration products instead of a continuous matrix were formed (Figure 4 and Figure A1). Therefore, the decrease in the volume fraction of the slag in the suspensions of SHAS and SWGAS even magnified the differences in the location of the growth of hydration products, emphasizing the role of the initial presence of soluble silicates during the hydration of AAS. Based on these results, one could expect a great effect of the slag volume fraction on the mechanical and other properties of SHAS, possibly higher than that seen with SWGAS.

During the first 24 h, the SCAS microstructure underwent the smallest changes with the used alkali activators (Figure 4), which again correlates with the calorimetric responses (Figure 1) and the generally observed long induction period. After 24 h, increased amounts of fine particles lying on the larger ones could be observed, but somewhat more representative is Figure A1, with a lower magnitude compared to Figure 4. Besides this, it appears that very fine slag particles (below 1 μm) slightly agglomerated or served as a nucleation site for the early reaction products of the SCAS. For these reasons, the BET surface area of the SCAS increased noticeably after 5 and 24 h (Table 3), although the formation of considerable amounts of C-A-S-H could not be expected at this stage. The agglomeration of the slag particles and well-developed crystals was magnified when the slag volume fraction was reduced to 0.10 (Figure A1). Hence, the volume fractions of fine particles of several microns determined using laser granulometry decreased considerably, while the contents of particles of 50–100 μm increased (Figure 3B). The last phenomenon that we would like to mention for the lower volume fraction of slag (0.10) in SCAS after 24 h is the roughened surface of the slag grains. This could be related to the increased dissolution at a lower volume fraction, or possibly from increases in C-A-S-H, as Bernal et al. [41] suggest that the main hydration stages of SCAS after carbonate anions are consumed are essentially carried out in an NaOH environment.

In contrast to the alkaline activators used, the slag in water did not show any observable changes in microstructure after 24 h, which also corresponds to the results already presented.

**Figure 6:**
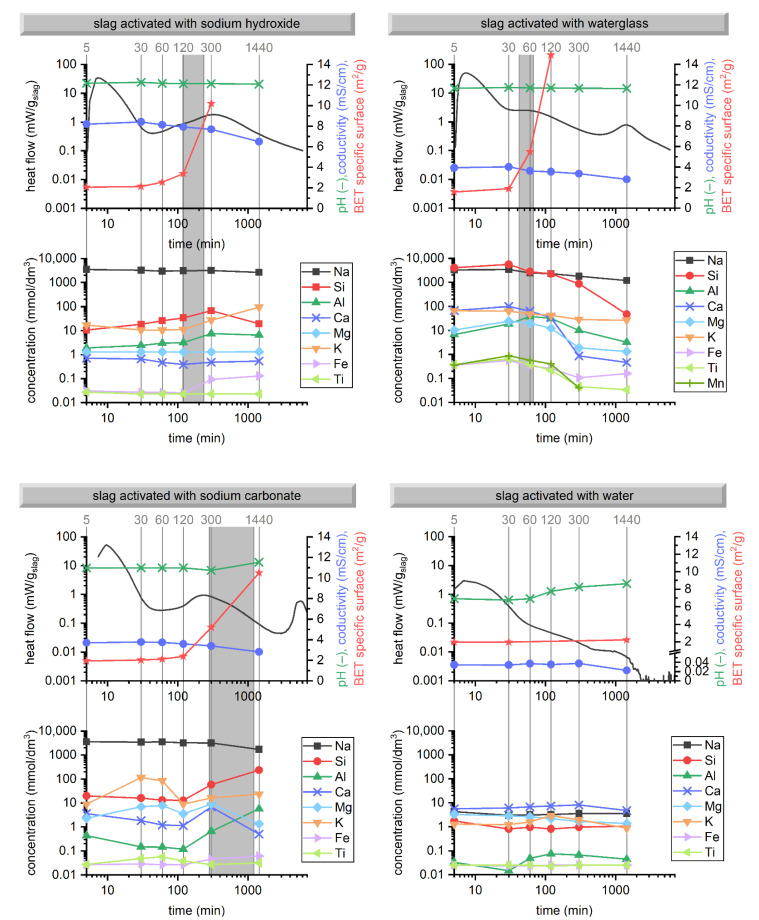
Correlations of pore solution composition, pH (100× diluted) and conductivity (100× diluted) with hydration kinetics, evolution of BET specific surface area and setting (grey area) of AAS depending on the activator type.

In addition to the investigations regarding the solid content of AAS, the pore solution composition was determined. After dilution of the pore solution (100× by volume), its pH and conductivity were also determined. To observe the evolution of these parameters over time for each activator and to compare the results for different activators, all results were put together and are given in Figure 6. Simultaneously, the already presented results on the hydration kinetics and setting are included. Data on the composition and properties of the pore solution are given in Table A2 (Appendix C).

For SHAS, the concentrations of network modifiers, namely Ca and Mg, were about 0.6 and 1.3 mmol/dm^3^, respectively, from the very early stages and throughout the first 24 h. Although slag contains about 4.5 times more Ca than Mg, the concentration of Ca was roughly half that of Mg. It should be noted that the Mg concentration was more stable, since all the measured points within the first 24 h fell into the range of 1.29 to 1.32 mmol/dm^3^, suggesting that it was driven by the same product throughout this stage. On the contrary, the Ca concentration started to decrease after 30 min and reached its minimum at a time similar to the initial setting time, which confirms the noticeable formation of hydration products (mainly C-A-S-H)—also observed using SEM (Figure 4 and Figure 5). Despite the precipitation of the hydration products, the concentrations of Si and Al during the first five hours still increased, which means that the dissolution of the slag aluminosilicate glass was faster than the consumption of the dissolved silicate and aluminate species. The precipitation of the hydration products was also related to the decrease in the conductivity of the pore solution, while the pH value remained roughly constant during the first 24 h. Both the conductivity and pH were determined for the 100× diluted solutions, and with respect to the high alkalinity of NaOH, the pH of the non-diluted pore solution should approach 14.

Significantly different was the situation of the pore solution composition in the case of activation with waterglass. In short, higher concentrations of all elements could be observed during the first minutes of hydration. Of course, the increased concentration of silicates was mainly due to their presence in the activating solution, but dramatically higher concentrations of cations originating from the slag suggest their accelerated release from the slag, which would be related to the lower pH observed compared to NaOH. Generally, higher concentrations of all elements tested were found for AASFA pastes with a higher silicate modulus compared to those with a lower silicate modulus (1.2 vs. 0.4) [65], which correlates with the higher concentrations observed in the present study, if the pore solution compositions of the sodium hydroxide and the sodium silicate-activated slag are compared. An increase in the concentrations of these elements in the presence of soluble silicates was also reported by Zuo et al. [66], who attributed it to the increased dissolution of Al from fly ash due to its complexation with soluble silicates. However, the results in the present work show the same phenomenon for sole AAS, indicating that higher Al concentrations were not limited to AAFA or AASFA. The concentrations of most elements started to decrease greatly between the 30th and 60th minute, resulting in the rapid formation of primary hydration products, responsible for the observed setting. Similar concentrations of the elements studied, as well as their sudden drop together with a sharp increase in the viscoelastic moduli of AASFA pastes, were reported by Dai et al. [65]. It is worth noting that the decrease in Al concentration started later than that of the other elements. This points to ongoing Al dissolution from slag and low Al incorporation in the silicate gel formed during these early stages, while Ca, Mg, and Na are incorporated. The changes in the pH values of the 100× diluted pore solution (11.65–11.71) were the smallest of all the investigated environments within the experimental error range, which could be related to the buffering effect of sodium silicate [1]. Similar to the SHAS, the conductivity slightly increased between 5 and 30 min and started to decrease as a consequence of the consumption of ionic species during the precipitation of the hydration products.

The evolution of the pore solution composition of the SCAS was noticeably different from that of the SHAS and SWGAS. The concentrations of Ca, Si, and Al noticeably decreased between the 5th and 120th minute, pointing to the precipitation of the hydration products. On the contrary, the concentrations of Mg and K initially increased, while their drop was evident at the 120th minute, after which they again increased. This also applies to Ca, Si, and Al. However, at 5 to 24 h, the concentrations of Ca and Mg decreased again, which was accompanied by a significant increase in pH and in the concentrations of Al and Si. These trends confirm the consumption of Ca and Mg due to their reaction with carbonates during the first hours and possibly days, which enabled further dissolution of the slag, including its aluminosilicate glass. A noticeable consumption of Na began after 60 min, which could be related to the formation of gaylussite or zeolite A, as both phases were determined [41] at the early stages of activation of the slag with sodium carbonate. 

The initial concentrations of Ca and Mg in the liquid phase of the slag suspension in water were similar to those determined in the SCAS, while the concentrations of Si and Al were approximately one order of magnitude lower, which is attributed to the relatively low pH of water. The 100× diluted pore solution was roughly neutral during the first 60 min of hydration, after which its pH started to increase. It is interesting that the concentration of Mg gradually decreased from the beginning of its measurement, while the concentration of Ca increased during at least the first 5 h, and was higher than that of Mg. Mg and Al were probably consumed for hydrotalcite formation, while Ca and Si formed C-S-H. Both of these hydration products were detected for the slag in water [67]. Decreased concentrations between 5 and 30 min could also be caused by their readsorption on the glass surface [60]. Lower concentrations of Mg compared to Ca, as well as their decreasing trend from the start of measurement, could be related to the very low solubility products of hydrotalcite-like phases [68]. Relatively high concentrations of Ca (even an order of magnitude higher compared to those in SHAS) may seem surprising, but are related to the relatively low pH of the pore solution—leading to the higher solubility of Ca^2+^ with respect to Ca(OH)_2_ [32], as well as very low concentrations of Al and Si, which could otherwise form C-A-H or C-A-S-H products. In general, the concentrations of all the elements were less than 10 mmol/dm^3^ during the first 24 h, indicating a relatively low degree of oversaturation, and thus only limited the formation of hydration products. The acceleration of the precipitation of hydration products started around 200 h, with a maximum around 300 h (see Figure 1, not included in Figure 6), but still reached only a limited extent. This illustrates the necessity of alkaline activators for inducing effective slag dissolution and the consequent formation of hydration products.

Up to now, the concentrations of minor elements, namely Fe, Ti, and Mg, have not been mentioned because they were close to or even below the detection limits and did not seem to play an important role in the process of alkali activation. Their concentrations seemed similar for SHAS, SCAS, and WAS, while they were found considerably higher for SWGAS—which corresponds well with higher concentrations of other elements that considerably decreased during the gelation stage. Finally, it is worth noting that the concentration of Fe seemed to increase after a certain time for all the AAS pastes. This could be related to the ongoing dissolution of slag, especially its aluminosilicate framework, as it is accompanied by an increase in the concentrations of Al and Si in the SHAS and SCAS pastes. A slight increase in Fe concentration could also be seen for the SWGAS between 5 and 24 h, which is again related to the dissolution of the slag and the resulting massive precipitation of C-A-S-H, manifested by the third calorimetric peak. At later stages beyond the first 24 h, the concentration of Fe would also decrease, as was observed in [66].

## 4. Conclusions

In this paper, the very early stages of AAS hydration were compared with respect to different activators (SWG, SH, and SC), in terms of changes in slag particles, evolution fresh-state properties, and the composition of the pore solution. In an effort to ensure the best comparability possible, all the activators had the same concentration of 4.0 mol Na^+^/dm^3^ and their dose was adjusted to set the volume fraction of slag in the whole paste to the same value of 0.50 (or 0.10 in some special cases). For comparison, a slag suspension in water was also prepared. Very distinctive behaviors were observed, monitored, and compared for all the pastes prepared.

SWGAS showed the lowest rheological properties just after mixing, but they increased quickly, and this paste set sooner than both other pastes due to the gelation of silicates from the activating solution, detectable mainly between the 30th and 120th minute of hydration. On the contrary, the slowest setting was observed for SCAS and was related to a stiffening of the paste due to the presence of carbonates and other products, rather than formation of the binding phase (C-A-S-H), as it was possible to disperse the paste in water even after 24 h.

The growth of the first hydration products around the setting stage could be effectively monitored by the BET specific surface area measurement. By far, the most rapid evolution in the specific surface area was observed for the SWGAS as a result of the gelation of silicates among the slag particles, as confirmed by SEM. Unlike the SWGAS, the SHAS was characteristic of the growth of the C-A-S-H being limited closer to the slag surface and with different morphology.

The anionic group of the activator dictated the pH, conductivity, and composition of the pore solution over time. In addition to the highest concentration of silicates introduced into the system by the activator itself, SWGAS showed the highest concentrations of all other determined species (Ca^2+^, Mg^2+^, K^+^, aluminates, minor elements) dissolved from the slag during the first 2 to 5 h of hydration. Following this, they were consumed or immobilized in the early reaction products. Very significant changes in the pore solution composition were also shown in the SCAS, mainly due to the presence of carbonate anions. After two hours, along with an increase in pH, the concentrations of Al and Si started to increase dramatically, which is important for the subsequent precipitation of C-A-S-H, which mainly occurs after 3–4 days.

The results obtained can serve to estimate the early behavior of AAS pastes with different activators based on their calorimetric curves or on other determined parameters, for comparison with other papers, and can contribute to the modeling of AAS hydration in the presence of different activators. In addition, knowledge of the composition of the pore solution as a key parameter affecting the behavior and compatibility of organic additives with these systems will help in the development of such admixtures for alkali-activated materials, which is still a pressing issue.

## Figures and Tables

**Table 1:** Chemical composition of the slag.

SiO_2_	Al_2_O_3_	Fe_2_O_3_	CaO	MgO	SO_3_	K_2_O	Na_2_O	TiO_2_	Mn_2_O_3_	SrO	LOI
39.4	8.08	0.73	37.0	8.56	1.36	1.18	0.40	0.30	0.88	0.06	2.0

**Table 2:** Initial and final setting time of AAS pastes with different activators (SWG—sodium waterglass, SH—sodium hydroxide, SC—sodium carbonate and W—water).

Activator	SWGAS	SHAS	SCAS	WAS
initial setting time	42 ± 2.8 min	118 ± 2 min	281 ± 4 min	>3 days
final setting time	67 ± 1.4 min	235 ± 7 min	1219 ± 11 min	>3 days

**Table 3:** BET specific surface area (m^2^/g) of the solid fractions isolated from the selected AAS pastes.

Activator	Original Slag	Time of Hydration					
		5 min	30 min	1 h	2 h	5 h	24 h
Water	1.85	1.98	1.98	n.d.	n.d.	n.d.	2.22
NaOH		2.06	2.13	2.54	3.37	10.2	n.d.
Sodium waterglass		1.57	1.90	5.50	14.9	n.d.	n.d.
Na_2_CO_3_		1.93	2.02	2.11	2.37	5.20	10.5

n.d. = not determined.

## Data Availability

The data presented in this study are available on request from the corresponding author. The data are not publicly available due to ongoing research.

## Appendix A

**Table A1:** Important points from the determination of the PSD of AAS pastes over time.

Sample	Time	x_10_	x_30_	x_50_	x_90_	x_99_	Modus	Geometric Mean
Original slag	–	1.63	6.16	12.57	38.37	62.42	21.18	16.74
WAS_0.50	5 min	1.54	6.04	12.49	38.22	62.09	22.05	16.64
	30 min	1.54	6.04	12.52	38.36	62.20	22.91	16.69
	1 h	1.54	6.05	12.49	38.12	61.77	22.91	16.62
	2 h	1.56	6.14	12.61	38.38	62.40	22.91	16.75
	5 h	1.53	5.99	12.39	37.90	61.54	22.05	16.51
	24 h	1.53	6.01	12.33	37.43	61.03	19.44	16.37
WAS_0.10	24 h	1.87	6.13	11.70	33.48	53.05	19.44	16.37
SHAS_0.50	5 min	1.52	6.07	12.58	38.12	61.38	22.91	16.65
	30 min	1.50	6.04	12.49	37.87	61.09	22.91	16.42
	1 h	1.50	6.00	12.45	37.82	60.97	22.91	16.51
	2 h	1.53	5.94	12.39	37.36	61.21	22.91	16.49
SWGAS_0.50	5 min	1.62	6.20	12.50	37.48	60.97	20.6	16.50
	30 min	1.60	6.14	12.42	37.04	60.84	19.44	16.37
SCAS_0.50	5 min	1.51	6.03	12.48	37.90	61.44	22.91	16.55
	30 min	1.51	6.02	12.49	37.82	60.90	22.91	16.52
	1 h	1.51	6.05	12.53	37.85	61.35	21.76	16.56
	2 h	1.51	6.13	12.76	38.36	61.90	22.91	16.78
	5 h	1.42	5.59	11.96	37.20	60.67	21.76	16.09
	24 h	1.43	5.43	11.83	37.03	60.80	21.76	15.99
SCAS_0.10	24 h	2.52	6.96	13.29	44.91	77.81	19.44	18.99

## Appendix C

**Table A2:** Compositions of the pore solutions (mmol/dm^3^), and their pH and conductivity κ (mS/cm) after a 100× dilution over time (min).

**Activation with Sodium Hydroxide**											
**Time**	**Na**	**Si**	**Ca**	**Mg**	**Al**	**K**	**Fe**	**Ti**	**Mn**	**pH**	**κ**
5	3481	10.42	0.7011	1.304	1.890	17.16	0.03044	0.02716	0.00546	12.17	8.202
30	3266	18.352	0.6562	1.304	2.394	10.56	0.02686	0.02298	0.00728	12.26	8.407
60	2989	25.91	0.4666	1.296	2.984	10.49	0.02686	0.02298	0.00364	12.16	8.126
120	3138	33.98	0.3892	1.292	3.187	11.19	0.02507	0.02298	0.00182	12.13	7.917
300	3194	67.53	0.4816	1.300	7.431	27.41	0.09132	0.02298	n.d.	12.13	7.695
1440	2647	19.28	0.5315	1.325	6.567	94.07	0.1289	0.02298	n.d.	12.08	6.486
**Activation with sodium waterglass**											
**Time**	**Na**	**Si**	**Ca**	**Mg**	**Al**	**K**	**Fe**	**Ti**	**Mn**	**pH**	**κ**
5	3327	4063	67.20	10.37	6.794	66.33	0.3528	0.3823	0.35859	11.67	3.928
30	3411	5631	98.81	24.46	18.72	64.65	0.5623	0.6539	0.89009	11.76	4.016
60	2500	2805	63.89	20.06	38.46	47.83	0.3313	0.3781	0.56973	11.71	3.624
120	2293	2275	34.02	12.40	31.83	42.88	0.2632	0.2215	0.40045	11.7	3.540
300	1794	862.5	0.8708	1.884	10.01	28.63	0.1057	0.04596	0.04005	11.68	3.368
1440	1189	47.17	0.4616	1.333	3.236	26.90	0.1594	0.03343	n.d.	11.65	2.806
**Activation with sodium carbonate**											
**Time**	**Na**	**Si**	**Ca**	**Mg**	**Al**	**K**	**Fe**	**Ti**	**Mn**	**pH**	**κ**
5	3529	19.84	3.603	2.279	0.4336	8.959	0.02686	0.02716	n.d.	10.97	3.698
30	3451	15.94	1.821	6.850	0.1483	113.1	0.02865	0.04805	n.d.	10.98	3.764
60	3520	13.14	1.193	7.838	0.1445	84.84	0.02686	0.05641	0.00182	10.99	3.739
120	3239	12.92	1.123	3.547	0.1186	8.939	0.02686	0.0376	n.d.	10.99	3.598
300	3169	58.22	6.734	8.866	0.6597	16.30	0.04477	0.02716	n.d.	10.74	3.397
1440	1711	232.8	0.5015	1.350	5.656	23.04	0.06088	0.03134	n.d.	11.53	2.827
**Activation with demineralized water**											
**Time**	**Na**	**Si**	**Ca**	**Mg**	**Al**	**K**	**Fe**	**Ti**	**Mn**	**pH**	**κ**
5	4.276	1.755	5.554	3.271	0.03336	1.276	0.02507	0.02507	n.d.	6.92	0.03437
30	2.997	0.8118	6.041	2.876	0.01482	1.215	0.02328	0.02507	n.d.	6.77	0.03404
60	3.149	0.9578	6.732	2.604	0.04818	1.576	0.02328	0.02507	n.d.	6.92	0.03718
120	3.236	0.8189	7.318	2.172	0.07412	2.918	0.02507	0.02298	n.d.	7.75	0.03515
300	3.636	0.9542	8.157	1.650	0.06671	1.944	0.02507	0.02507	n.d.	8.25	0.03728
1440	3.589	1.050	4.713	1.358	0.04447	0.8722	0.02507	0.02507	n.d.	8.63	0.02247

n.d. = not determined.
